# The quality of life after trans oral video-assisted thyroidectomy and cervical thyroidectomy: a systematic review and meta-analysis

**DOI:** 10.3389/fsurg.2023.1116473

**Published:** 2023-05-17

**Authors:** Ibrahim A. Altedlawi Albalawi, Hyder Osman Mirghani

**Affiliations:** ^1^ Department of Surgery, Faculty of Medicine, University of Tabuk, Tabuk, Saudi Arabia; ^2^ Department of Internal Medicine, Faculty of Medicine, University of Tabuk, Tabuk, Saudi Arabia

**Keywords:** oral thyroidectomy, transcervical thyroidectomy, vestibular approach, quality of life, cosmetic thyroidectomy

## Abstract

Trans oral video-assisted thyroidectomy (TOVAT) is increasingly performed for cosmetic reasons. The quality of life after thyroidectomy is important for decision-making. This is the first meta-analysis to compare the quality of life among conventional transcervical thyroidectomies. This meta-analysis aimed to assess the same in the current literature. The authors systematically searched PubMed, Google Scholar, and EBSCO for relevant articles from the first published to December 4, 2022. The keywords endoscopic transoral via vestibular thyroidectomy, transcervical thyroidectomy, conventional thyroidectomy, scarless thyroidectomy, and quality of life were used. Out of the 482 studies retrieved, 27 full texts were reviewed, and only six fulfilled the inclusion and exclusion criteria. Patients with transoral thyroidectomy showed better quality of life that their counterparts who underwent transcervical thyroidectomy at 4–6 weeks following surgery, odd ratio, 2.26, 95% CI, 2.02–2.5, *P*-value <0.001. Substantial heterogeneity was observed, *I*^2^ for heterogeneity, 100%. The quality of life was better among patients who underwent the trans oral video-assisted thyroidectomy (TOVAT) compared to their counterparts with the conventional cervical approach (surgical questionnaire). All the components of the SF-36 quality of life questionnaire were better among TOVAT compared to the conventional approach except for social and general health components, which were equal between the two arms. Further multi-center studies with larger samples and controlling for pain and the surgical curve are needed.

## Introduction

Thyroid surgery is increasingly performed mirroring the increasing detection of low-risk differentiated thyroid carcinoma. Transoral thyroidectomy through different approaches is more popular due to the lack of unwanted neck scars (1). In addition, to the minimal dissection needed and surgical anatomical space respect. The patients are selected depending on thyroid diameter, thyroid volume, and underlying thyroid disease (2). Endoscopic thyroidectomy through the sublingual and the trans-tracheal approaches was first performed in Germany for a better cosmesis and discontinued due to safety issues (3, 4). Transoral thyroidectomy had the same outcomes as the traditional transcervical approaches with better cosmetic outcomes. However, unusual complications including mental nerve injury, carbon dioxide embolism, infections, burns, and perforation (5) are of great concern. Due to the excessive emphasis on physical appearance especially among young females and those prone to excessive scarring and keloids. Remote approaches including transoral thyroidectomy (either robotic or endoscopic approaches) are gaining popularity among both patients and Surgeons (6). Trans oral video-assisted thyroidectomy is increasingly used as truly scarless thyroid surgery for low-risk differentiated thyroid carcinoma. Patients who underwent thyroidectomy were found to live longer and have a lower quality of life compared to the general population (7, 1). Literature comparing different forms of remote thyroidectomy is scarce, and this is the first meta-analysis to compare transoral endoscopic thyroidectomy and conventional thyroidectomy. Therefore, this meta-analysis aimed to compare the quality of life of transoral and conventional thyroidectomy.

## Subjects and methods

### Eligibility criteria according to PICOS

The studies were included if they were retrospective or prospective cohorts, case-control studies, or randomized controlled studies (RCTs) comparing the quality of life among patients who underwent thyroidectomy via the conventional cervical approach or the endoscopic transoral via vestibular route. Thyroidectomy via other routes including areolar, breast axillary, and postauricular was not included. Case reports and case series were not included.

### Outcome measures

Comparing the quality of life between conventional thyroidectomy and trans oral video-assisted thyroidectomy.

### Information sources and search

The two researchers searched three databases, PubMed, Medline, EBSCO, and Google Scholar. The search engine was limited to the period from the first published article up to December 2022. The keywords trans oral video-assisted thyroidectomy, transcervical thyroidectomy, conventional thyroidectomy, scarless thyroidectomy, and quality of life were used.

**Figure 1:**
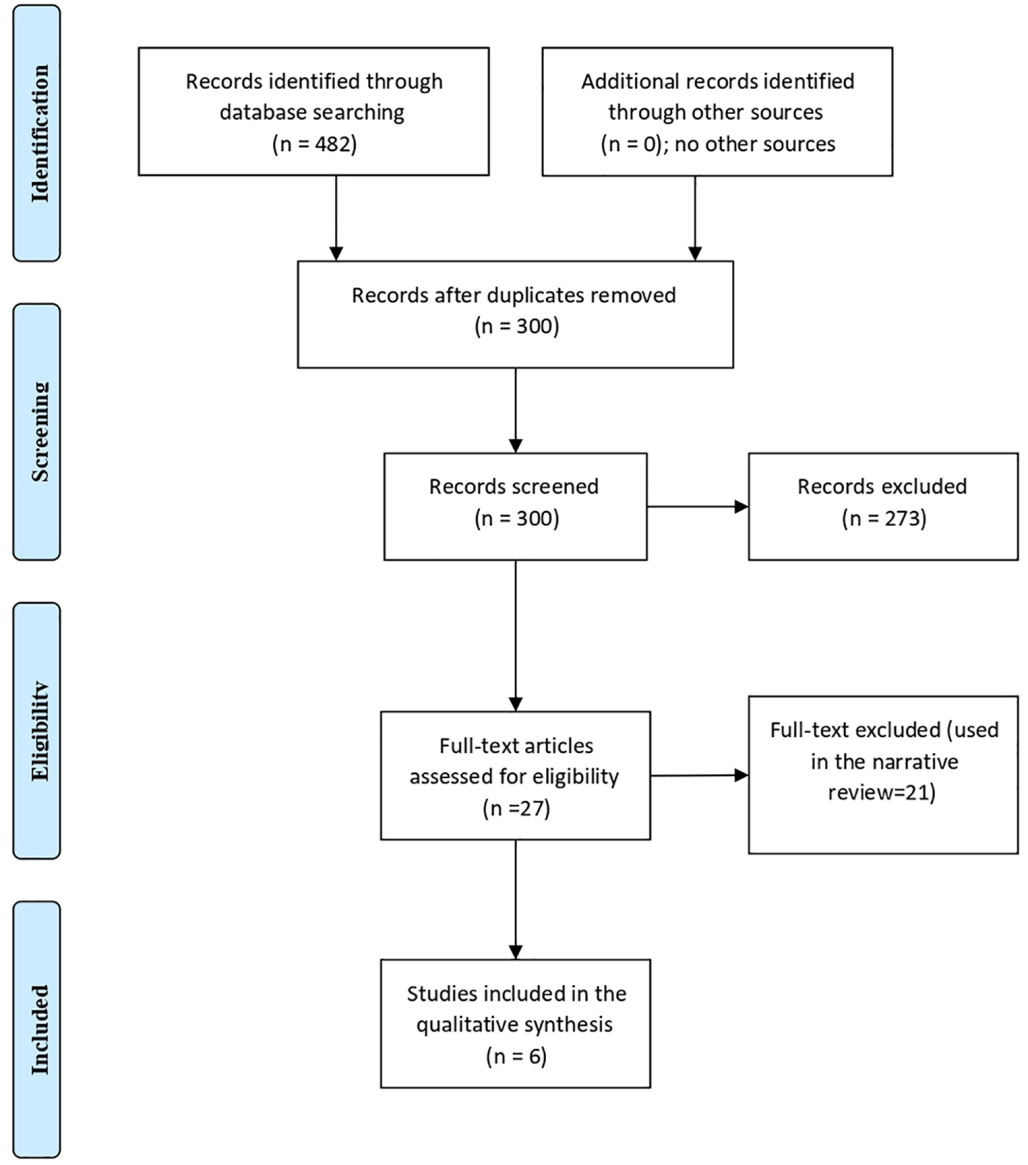
A comparison between the endoscopic trans oral video-assisted thyroidectomy and conventional thyroidectomy quality of life (the PRISMA chart).

The information retrieved included the author's name, year of publication, patient's number, the study duration, the criteria of patient selection, the quality of life using the SF-36, SF-12 quality of life questionnaire, and surgical questionnaire. The two authors cross-checked the data and discrepancies were solved by consensus. Newcastle Ottawa Scale assessed the quality of the included study (8) (Figure 1 and Tables 1, 2).

**Table 1:** Characteristics of the included studies.

Author	Country	Age	Females	Benign nodules	Quality of life
Johri et al. (9)	India	30.7 ± 9.2 vs. 33.0 ± 9.6	90.2% vs. 75%	100% in all	ThyPRO-39hin score
Kasemsiri et al. (10)	Thailand	38.3 ± 11.3 vs. 46.7 ± 10.9	100% vs. 89.5%	96.9% vs. 100%	SF-36 and Surgical
Nguyen et al. (11)	Vietnam	38 ± 10.5 vs. 52.5 ± 13.4	97.9% vs. 90.3%	100% in all	Satisfaction level
Van Den Heede et al. (12)	France	40 vs. 51	100% vs. 76%	82% vs. 63%	SF-12 and surgical
Xuan Nguyen et al. (13)	Vietnam	35.8 + 10.3 vs. 46.9 + 11.5	90% vs. 88.5%	22.3% vs. 26.2%	SF-36 and Surgical
Alnehlaoui et al. (14)	United Arab Emirates	42.37 ± 9.33 vs. 44.65 ± 10.68	83.8% vs. 67.8%	71% vs. 32%	SF-36 questionnaire

**Table 2:** Quality assessment of the included studies.

Reference	Selection	Compatibility	Outcome	Overall
Johri et al. (9)	3	1	3	7
Kasemsiri et al. (10)	3	1	2	6
Nguyen et al. (11)	3	1	2	6
Van Den Heede et al. (12)	3	1	2	6
Xuan Nguyen et al. (13)	3	1	2	6
Alnehlaoui et al. (14)	3	1	3	7

#### Data analysis

The RevMan system for meta-analysis was used, and the data were all continuous. The fixed effect or random effect was used depending on the level of heterogeneity. Funnel plots were used to assess lateralization. A *P*-value of <0.05 was considered significant.

## Results

### Characters of the included studies

There were six studies (9–14), five from Asia and one from France. Patients with TOETVA were younger and mostly females, all patients were diagnosed with benign thyroid nodules in three studies, with more benign nodules among patients with TOETVA in the other three studies.

**Figure 2:**
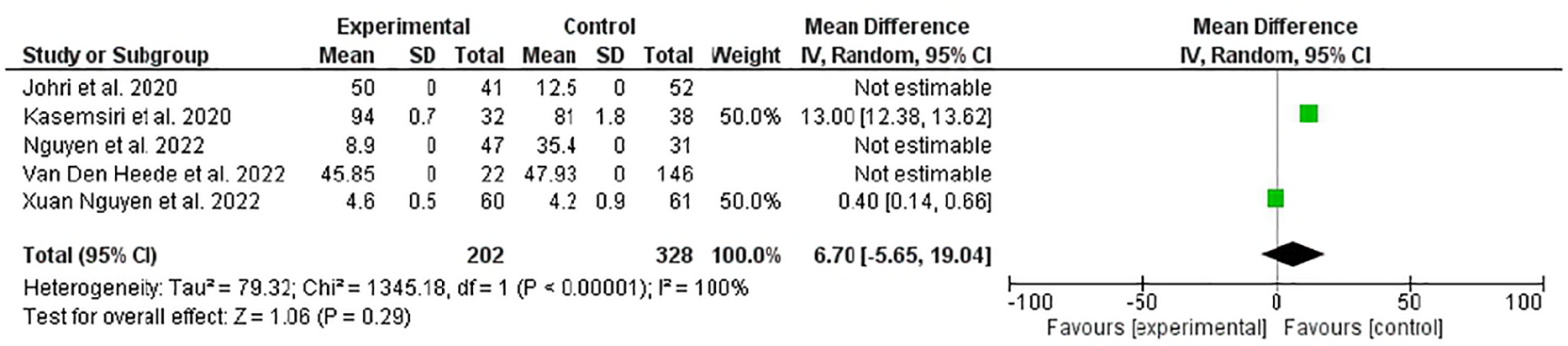
A comparison of quality of life between trans oral video-assisted thyroidectomy and conventional open thyroidectomy at 4–6 weeks (specific thyroid surgery-related QOL questionnaire).

In the present meta-analysis patients with transoral thyroidectomy showed better quality of life that their counterparts who underwent transcervical thyroidectomy at 4–6 weeks following surgery, odd ratio, 2.26, 95% CI, 2.02–2.5, *P*-value <0.001. Substantial heterogeneity was observed, *I*^2^ for heterogeneity, 100%. Figure 2 showed the quality of life of 530 patients.

**Figure 3:**
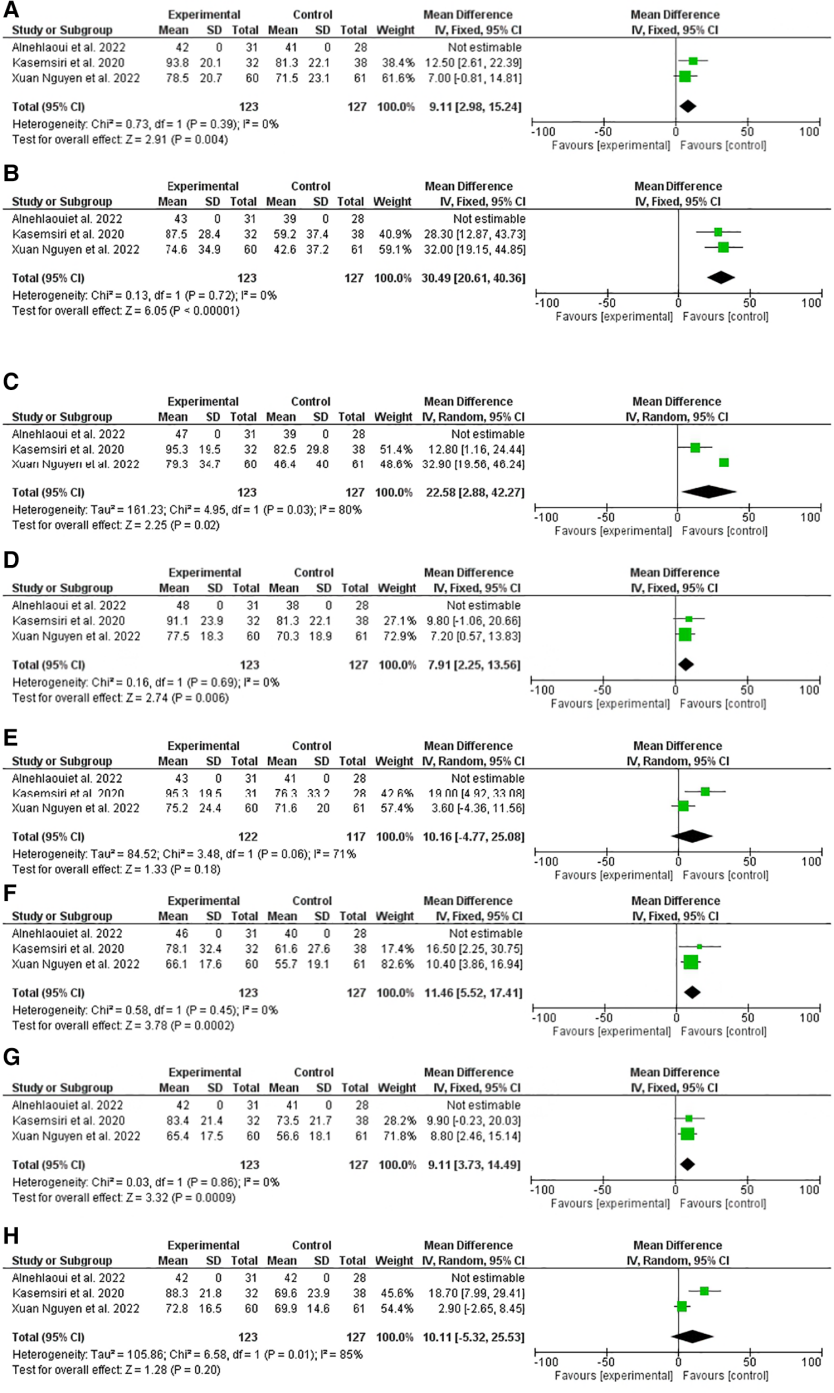
A comparison of quality of life between trans oral video-assisted thyroidectomy and conventional open thyroidectomy at four-six weeks after surgery (36- item short-form health survey). (**A**) Physical function, (**B**) role physics, (**C**) emotion, (**D**) bodily pain, (**E**) vitality, (**F**) social function, (**G**) mental health, (**H**) general health.

In the present meta-analysis, three studies including 322 patients were pooled. The quality of life was assessed using the thyroid surgery-specific questionnaire. Transoral thyroidectomy showed a better physical function compared to conventional thyroidectomy, odd ratio, 9.11, 95% CI, 2.98–15.24, no heterogeneity was observed, *I*^2^, 0.0, *P*-value for overall effect, 0.004. Regarding role physics, TOVAT showed better results, odd ratio, 30.49, 95% CI, 20.61–40.36, no heterogeneity was observed, *I*^2^, 0.0%%, *P*-value for overall effect <0.001. Emotion, bodily pain, and vitality were better among the TOETVA arm, with odd ratio, 22.58, 95% CI, 2.88–42.27, odd ratio, 7.91, 95% CI, 2.25–13.56, odd ratio, 11.46, 95% CI, 5.52–17.41. However, no difference was evident regarding social function, odd ratio, 10.16, 95% CI, 4.77–25.8. Transoral thyroidectomy was better than conventional thyroidectomy regarding mental health, but not general health, odd ratio, 9.11, 95% CI, 3.73–14.49, and odd ratio, 10.11, 95% CI, 5.32–25.53, respectively, *P*-values, 0.009, and 0.2 respectively (Figure 3).

## Discussion

Patients with papillary thyroid carcinoma (especially young women) are willing to travel and pay extra expenses to avoid the visible open thyroidectomy scar. Trans oral video-assisted thyroidectomy (TOVAT) is a scar-free approach that satisfies the cosmetic requirements of the patients. Due to the long survival of PTC, many patients including the elderly are more concerned about cosmetic outcomes and quality of life (15, 16). In the present meta-analysis, TOVAT was associated with a better quality of life at 4–6 weeks compared to conventional thyroidectomy using specific thyroid surgery-related QOL questionnaire overall satisfaction (odd ratio, 2.26, 95% CI, 2.02–2.5). However, discrepancies were observed among the different items of 36- the item Short-Form Health Survey. TOVAT was better in all quality of life domains except for the social and general health domains in them there was no difference between the two approaches. A plausible explanation might be the SF-36 is not specific in evaluating cancer symptoms (17). In addition, the quality of life among patients with papillary thyroid carcinoma differs significantly according to the temporal profile (worse at one month and improves in six months but never reaches that of the general population) (18). Furthermore, patients with thyroid cancer tend to have poor role emotion for years after surgery; this is because they fear the rare rate of metastasis (19). Absence of scaring improved the cosmetic outcomes, overall satisfaction, and quality of life among patients with TOVAT compared to their counterparts who underwent open surgery. Importantly, TOVAT might improve communication, career development, self-esteem, quality of life, and fashion (20–22). Postoperative exercise can improve pulling sensation and limitation of neck movement from corridor application in TOVAT. Therefore, early neck exercises are recommended to improve the quality of life among patients undergoing TOVAT (23).

In the present meta-analysis, most of the patients who choose TOVAT were young females with benign thyroid nodules. The explanations are that young females are more concerned about cosmetic appearance, also, the extent of surgery and the Surgeon's experience might affect the operation choice (24).

Importantly, low/intermediate grade differentiated thyroid carcinoma are heterogeneous. Tumor-specific genetic alterations and vascularization are linked to recurrence and prognostication (many germline vascular endothelial growth factor-A single nucleotide polymorphisms) (25). It is vital to consider the above possibility when choosing the approach for thyroidectomy as TOVAT might increase the seeding of such tumors. Another important issue is the calcitonin negative tumors, which pose a clinical diagnostic challenge (26). Recurrent laryngeal nerve palsy is the most feared post-thyroidectomy complications with medicolegal litigation; transcutaneous laryngeal ultrasonography is a noninvasive sensitive measure for evaluating laryngeal nerves before operation with a high concordance to laryngoscopy (27).

The study had several limitations: the small number of the included studies, the short duration of the studies included, not reporting the analgesia that might affect the quality of life, and not evaluating the quality of life before surgery.

## Conclusion

The quality of life was better among patients who underwent the trans oral video-assisted thyroidectomy compared to their counterparts with the conventional cervical approach (surgical questionnaire). All the components of the SF-36 quality of life questionnaire were better among TOVAT compared to the conventional approach except for social and general health components, which were equal between the two arms. Further multi-center studies with larger samples and controlling for pain and the surgical curve are needed.
